# Exploring Early Pre-Symptomatic Detection of Influenza Using Continuous Monitoring of Advanced Physiological Parameters during a Randomized Controlled Trial

**DOI:** 10.3390/jcm10215202

**Published:** 2021-11-08

**Authors:** Nir Goldstein, Arik Eisenkraft, Carlos J. Arguello, Ge Justin Yang, Efrat Sand, Arik Ben Ishay, Roei Merin, Meir Fons, Romi Littman, Dean Nachman, Yftach Gepner

**Affiliations:** 1 Department of Epidemiology and Preventive Medicine, School of Public Health, Sackler Faculty of Medicine, and Sylvan Adams Sports Institute, Tel-Aviv University, Tel-Aviv 6997801, Israel; nir@bio-beat.com (N.G.); gepner@tauex.tau.ac.il (Y.G.); 2 Biobeat Technologies LTD, Petah Tikva 4951122, Israel; sand@bio-beat.com (E.S.); arik@bio-beat.com (A.B.I.); m.roei@bio-beat.cloud (R.M.); meir@bio-beat.com (M.F.); romi@bio-beat.cloud (R.L.); 3 The Institute for Research in Military Medicine, The Hebrew University Faculty of Medicine, The Israel Defense Force Medical Corps, Jerusalem 9112102, Israel; deannahman@gmail.com; 4 Leidos, Reston, VA 20190, USA; Carlos.ArguelloOrtiz@hhs.gov; 5 Department of Health and Human Services, Biomedical Advanced Research and Development Authority (BARDA), Washington, DC 20201, USA; Ge.Yang@hhs.gov; 6 Heart Institute, Hadassah Medical Center, The Hebrew University of Jerusalem, Jerusalem 9112102, Israel

**Keywords:** remote patient monitoring, physiological patterns, bio-surveillance, biological outbreak, influenza, photoplethysmography

## Abstract

Early detection of influenza may improve responses against outbreaks. This study was part of a clinical study assessing the efficacy of a novel influenza vaccine, aiming to discover distinct, highly predictive patterns of pre-symptomatic illness based on changes in advanced physiological parameters using a novel wearable sensor. Participants were frequently monitored 24 h before and for nine days after the influenza challenge. Viral load was measured daily, and self-reported symptoms were collected twice a day. The Random Forest classifier model was used to classify the participants based on changes in the measured parameters. A total of 116 participants with ~3,400,000 data points were included. Changes in parameters were detected at an early stage of the disease, before the development of symptomatic illness. Heart rate, blood pressure, cardiac output, and systemic vascular resistance showed the greatest changes in the third post-exposure day, correlating with viral load. Applying the classifier model identified participants as flu-positive or negative with an accuracy of 0.81 ± 0.05 two days before major symptoms appeared. Cardiac index and diastolic blood pressure were the leading predicting factors when using data from the first and second day. This study suggests that frequent remote monitoring of advanced physiological parameters may provide early pre-symptomatic detection of flu.

## 1. Introduction

Influenza (flu) viruses spread easily from person to person by droplet transmission, initiating a quick onset of the disease. Clinically, influenza causes a wide range of clinical signs and symptoms ranging from non-febrile, mild upper-respiratory-tract infection that resolves within up to two weeks to severe or even fatal complications such as pneumonia and sepsis [1]. Influenza patients often exhibit some or all of the following symptoms: fever, chills, cough, sore throat, rhinorrhea, muscle aches, headaches, and fatigue. Influenza can also worsen and complicate asthma, congestive heart failure, pregnancy, morbid obesity, and various other chronic medical conditions [2,3,4,5,6]. Pandemics of influenza can cause catastrophic illness and societal disruption and consequently rank high among natural threats that necessitate ongoing public health and medical preparedness [7]. Several influenza pandemics have appeared since the beginning of the 20th Century [8,9,10,11,12] and recently it was shown that a 24-h delay in identification significantly increases the odds of death [6].

It is accepted that vital signs during the acute phase of flu, such as blood oxygen saturation (SpO_2_), heart rate (HR), and respiratory rate (RR) show changes, usually decrease in SpO_2,_ and increase in HR and RR, yet it is difficult to rely on them as changes are not specific enough, measurement rate is low, and there are no defined patterns characteristic of influenza [13]. Patient characteristics and local surveillance data on influenza outbreaks are important for differential diagnoses and management decisions, owing to differences in clinical outcomes [14]. Traditional bio-surveillance used to detect, inspect, and respond to disease outbreaks has focused on retrospectively gathering and monitoring diagnostic medical and public health data to determine the existence of a disease outbreak [15]. Usually, the information is collected, delivered, and analyzed days, weeks, or even months after the outbreak, and by the time it reaches decision-makers, it may be too late for public health interventions to avoid early cases or to put prevention methods into place.

The distribution of wearable monitors in different settings as an early detection tool, supporting syndromic surveillance during suspected outbreaks, could provide significant advantages and improve timely healthcare delivery [16,17]. Moreover, it is postulated that using wearable devices for continuous physiological monitoring could potentially allow prediction and early warning before severe emergencies such as mass biological outbreaks occur [16,17,18].

This controlled study aimed to monitor healthy subjects who received a novel influenza virus vaccine based on modified vaccinia virus ankara (MVA-NP + M1) using wearable remote patient monitoring devices, looking for potential physiological patterns during the early course of the disease.

## 2. Materials and Methods

### 2.1. Study Design and Overview

This monitoring study was conducted between May 2019 and January 2020 within the framework of a Phase 2, single-center, randomized, double-blinded study evaluating the safety, efficacy, and immunogenicity of a novel flu vaccine in healthy volunteers (NCT03883113). It consisted of an outpatient vaccination phase followed by an inpatient challenge phase two months later. Both the research and the analyzing teams were blinded as to which vaccination was administered to each subject. For the full duration of the study the subjects, including healthy male and female volunteers, were using a wearable wrist-monitor (BB-613WP, Biobeat Technologies Ltd., Petah-Tikva, Israel) to collect and record their physiological parameters. The physiological data obtained by the devices were not available to any of the researchers on-site and were analyzed retrospectively. The study was approved by the local Institutional Review Board (Commissie voor Medische Ethiek ZNA, EC Approval Number 5238; the date of registration for the clinical trial was 17 April 2019). All participants signed an informed consent form before the procedure. The study was conducted in a confined research facility (SGS Life Sciences, Clinical Pharmacology Unit, Antwerp, Belgium).

### 2.2. Study Procedures and Interventions

**Figure 1:**
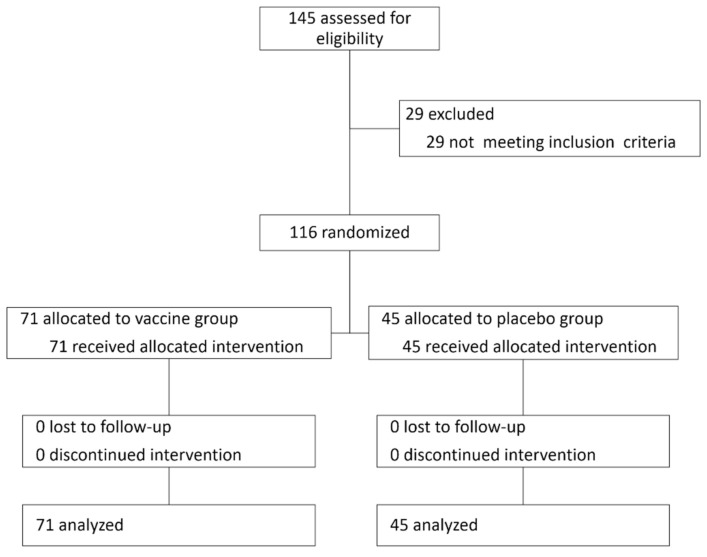
The CONSORT diagram with participants’ flow.

**Figure 2:**
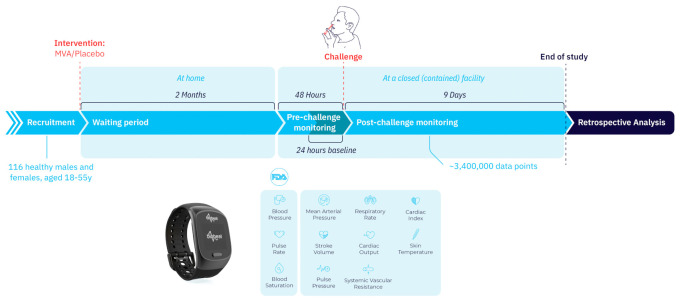
Study design. A total of 116 healthy individuals were recruited. Two months following randomization and treatment with vaccine or placebo, they were admitted into a confined facility in which they were continuously monitored using an FDA-cleared wireless remote patient monitoring device. 48 h after admission all were intranasally exposed to H3N2 influenza virus, monitoring continued for 9 more days, and daily surveys were completed twice a day during the whole 11 days. The physiological parameters were collected automatically with no personally identifiable information and analyzed retrospectively following study completion.

Participants were randomly assigned into vaccine (MVA-NP + M1) or placebo (saline) groups, injected intramuscularly. Two months after intervention they were all challenged intranasally using a Teleflex VaxINator^TM^ kit with the H3N2 human influenza virus (Figure 1 and Figure 2).

Continuous monitoring of 12 physiological parameters started 24–48 h prior to virus inoculation and during the following 9 days. For all measured parameters, the baseline period was defined as the mean value for each participant during the 24 h before exposure. Physiological parameters were automatically measured for each subject every 5 min throughout the whole study period using the wrist-monitors. These parameters included HR, SpO_2_, RR, systolic blood pressure (SBP), diastolic blood pressure (DBP), mean arterial pressure (MAP), pulse pressure (PP), stroke volume (SV), cardiac output (CO), cardiac index (CI), systemic vascular resistance (SVR), and temperature. The collected data had no personal identifiable information (PII) besides serial numbers of the devices.

### 2.3. The Wearable Monitoring Devices

The medical-grade monitoring devices used in the study employ reflective photoplethysmography (PPG) technology, capturing changes in tissue reflectance and measuring numerous vital signs including advanced hemodynamic parameters [19,20,21,22,23]. More specifically, the waveform is related to the reflection caused by the blood vessels when they change diameter. The high resolution of the PPG wave alongside advanced algorithms analyze the pulse wave transit time (PWTT) and provide pulse wave analysis (PWA) which allows the sensor to track vital signs as well as to capture changes, derived from the pulse delineations. Data are transmitted through Bluetooth low energy (BLE) to a dedicated gateway, and from the gateway, the data is transmitted through either Wi-Fi, cellular, or ethernet to a cloud-based repository, for final analysis.

The wrist monitors require a single trimonthly calibration of the pulse rate (PR) and blood pressure (BP) baseline using an approved non-invasive cuff-based device. Within the context of this study, calibration was performed only once at the beginning of the monitoring period. The wrist monitor is FDA-cleared for heart rate, SpO_2_, and non-invasive cuffless BP measurements and has a CE mark approval for all vitals. In this study, all vitals were monitored and included in the analysis. The calibration process starts with the individual sitting at rest, as defined by international guidelines on blood pressure measurement. After assuring the cuff size is appropriate for the individual’s arm, a measurement is taken using an approved cuff-based device. This is repeated three times, and the average of the three measurements is entered as the baseline calibration value. This is the same for the heart rate value. The temperature is measured using the “double-sensor” technique with thermistors included within the sensor unit.

### 2.4. Laboratory Tests

Nasal swabs for influenza virus were collected twice a day (at least 8 h apart) from day 2 to day 10 following the intranasal challenge, tested for influenza, PCR, and culture. Viral load was measured by qPCR and virus titration assay, with detection threshold defined as 2.18 log10 viral particles/mL for the qPCR and 0.75 log10 TCID 50/mL for the virus titration assay (defined as the lowest threshold for which these tests can be considered reliable). A positive for influenza was defined with at least two consecutive positive swabs as determined by qPCR and culture, respectively.

### 2.5. Daily Questionnaires

Participants were asked to fill out questionnaires twice a day, in the morning and in the evening. They were filled up to 11 days after the challenge. The questionnaire included questions on medications taken during the last 12 h, presence of lymphadenopathy, whether they feel well enough to work, whether there are signs of upper respiratory tract infection (including nasal congestion, runny nose, sneezing and its frequency, sinus pressure, or facial pain, sore throat, difficulty swallowing, teary or watery eyes, painful eyes (aversion to light), whether there are signs of lower respiratory tract infection (dry cough, productive cough, difficulty in breathing), gastrointestinal complaints (nausea, stomach ache, vomiting, diarrhea), and general complaints (malaise, dizziness, head congestion, headache, muscle or joint aches, fever, chills, shivering, lack of appetite, feeling cold, sweating, tiredness or weakness, sleeping more than usual, fatigue, or itching).

### 2.6. Statistical Analysis

All data are presented as mean ± standard deviation. Between-groups comparison was carried out using independent samples *t*-test. Special attention was given to subgroups of sex and age, looking for a potential difference in the physiological response to flu between males and females, as well as between different age groups. For the comparison of changes in the physiological parameters over time, we used mixed-model ANOVA to evaluate time-treatment, time-sex, time-age, and time-detected virus interactions. For between-days comparison, we used repeated-measures ANOVA, and in cases where significant difference was achieved, Tukey’s posthoc test was used. Pearson correlation was used to evaluate the association between changes in the measured parameters and virus levels. Significance was achieved for *p*-value below 0.05. Data analysis visualizations were made using Pingouin (repeated-measures and mixed ANOVA tests, and for interaction assessment) [24], SciPy stats (independent samples t-test) [25], Scikit-posthocs (Tukey posthoc tests) [26], Scikit-learn (machine learning models) [27], Imblearn (SMOTE) [28], and Seaborn (visualization) [29].

### 2.7. Classification Model

Four classification models (logistic regression, K-nearest neighbors, kernel support vector machine, and random forest classifier) were tested for their ability to classify participants as flu positive or negative, based on the mean daily change from the 24-h baseline period in the measured physiological parameters that were continuouly monitored by the wearable devices. All measured parameters were used as a feature in the model. Due to the imbalanced dataset, the minority class was scaled up by creating synthetic samples using the synthetic minority oversampling technique (SMOTE) method [30] to achieve equal proportions among classes. The data set was stratified into training and validation using repeated K-fold 4 cross-validation (splits −75% of the data for training and 25% for testing, with 3 repeats for a total of 12 combinations) to increase the confidence in the results. SMOTE was used inside the cross-validation process, for each fold. Model performance was evaluated and the following parameters were calculated: recall, precision, f1-score, and the avarage precision, which are more appropitae to imbalanced data [31]. The model with the highest overall score was selected for the calculation and analysis of feature importance.

## 3. Results

**Figure 3:**
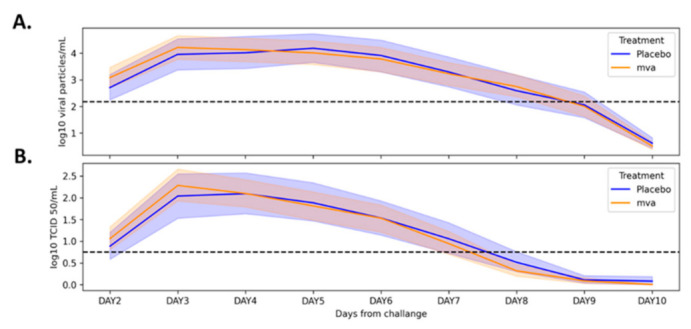
Changes in viral load following exposure among placebo- and MVA-treated groups. Mean (±95% confidence interval) viral load as measured by real-time PCR (**A**) and titration assay (**B**) throughout the study period. MVA: modified vaccinia virus ankara.

In this double-blinded, controlled study, 116 participants (67 females) were included in the analysis out of 145 participants recruited to the vaccine study (see attached Figure 1 with a CONSORT diagram and Figure 2), with a mean age of 40.2 ± 10.5 years. 71 participants (61%) received the vaccine and 45 received the placebo. No significant differences were observed in baseline characteristics between the treatment groups (Table 1). In both groups, virus levels peaked three days following the challenge and declined from then on (Figure 3A,B). In 11 participants (10 from the MVA group and 1 from the placebo group), virus levels in the routine laboratory tests collected were under the detection threshold, and they were regarded as flu-negative. Within the scope of the vaccine study, the novel flu vaccine did not confer the anticipated protection, with 90.1% of MVA-treated individuals with positive qPCR and 77.5% with a positive culture.

**Figure 4:**
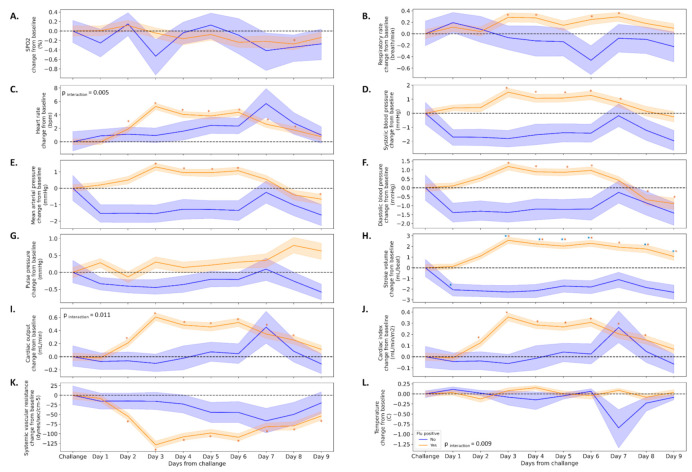
Changes in physiological parameters following exposure among participants in which virus was detected and among participants in which virus was not detected. Relative changes from baseline in physiological parameters (**A**–**L**) among participants in which virus was detected (blue lines) or not detected (orange lines). Dashed line represents the baseline. The interaction was assessed using mixed-model ANOVA. * Value significantly (*p* < 0.05) different from baseline.

We divided the study cohort into two groups based on the qPCR and the virus titration assay (*n* = 105 positive, *n* = 11 negative) and analyzed the mean changes from baseline for all 12 physiological parameters. In parallel to the increase in viral load, the majority of physiological parameters were increased, such as HR (5.3 ± 11.9 beats per minute), SBP (1.5 ± 7.4 mmHg), MAP (1.3 ± 5.5 mmHg), SV (2.6 ± 7.5 mL), and SVR (−128.7 ± 239.6 dynes.s.cm^−5^) three days following the infection. This pattern appeared only among participants in the flu-positive group (Figure 4). Interactions were observed in several parameters such as HR, temperature, and cardiac output (Figure 4C,I,L). Among the flu-negative participants, SV was the only parameter that significantly decreased (−2.3 ± 4.4 mL) from baseline values.

**Figure 5:**
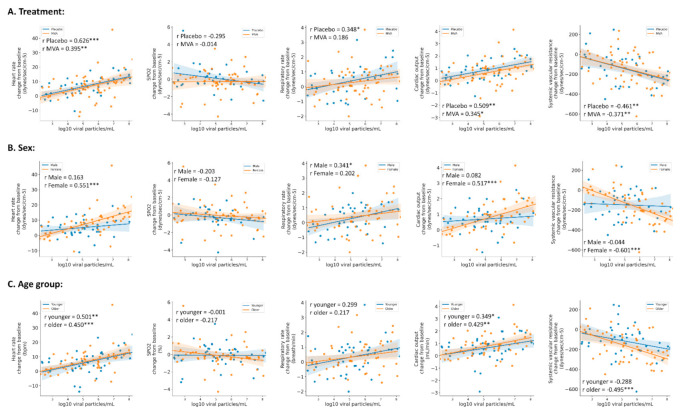
Correlations between viral load and changes in physiological parameters during the third-day post-exposure. Pearson’s correlations between viral loads as measured by real-time PCR and mean changes in physiological parameters from the third-day post-exposure. The correlations were stratified based on treatment ((**A**) Placebo, blue dots/line; MVA, orange dots/line), sex ((**B**) male, blue dots/line; female, orange dots/line), and age ((**C**) younger, blue dots/line; older, orange dots/line). * *p* < 0.05, ** *p* < 0.01, *** *p* < 0.001.

Next, we looked at whether the changes in the measured parameters were associated with changes in the viral load tests. We found that for the entire population, the highest correlations were achieved on the third day between RT-PCR results and changes in HR (*r* = 0.516), CO (*r* = 0.482), and RR (*r* = 0.286), *p* < 0.001 for all. We therefore analyzed the correlations on the third day among the different groups—treatment (placebo vs. MVA), sex, and age (Figure 5). Similar trends were observed between placebo- and vaccine-treated participants (Figure 5A). In addition, stronger correlations were achieved between RT-PCR results and changes in HR (*r* = 0.551 and 0.450), CO (*r* = 0.517 and 0.429), and SVR (*r* = −0.601 and −0.495) in females and in older participants (Figure 5B,C, respectively), while among males and young participants, a stronger association (*r* = 0.348 and 0.341) was achieved with changes in RR, respectively.

Finally, we analyzed pre-symptomatic physiological changes in all measured parameters among all participants using four classification models: logistic regression, k-nearest neighbors, kernel support vector machine and random forest. This analysis aimed to define the physiological pattern of changes characterizing this pre-symptomatic phase of flu, to help in the development of a future predictive tool which might be used for bio-surveillance.

**Figure 6:**
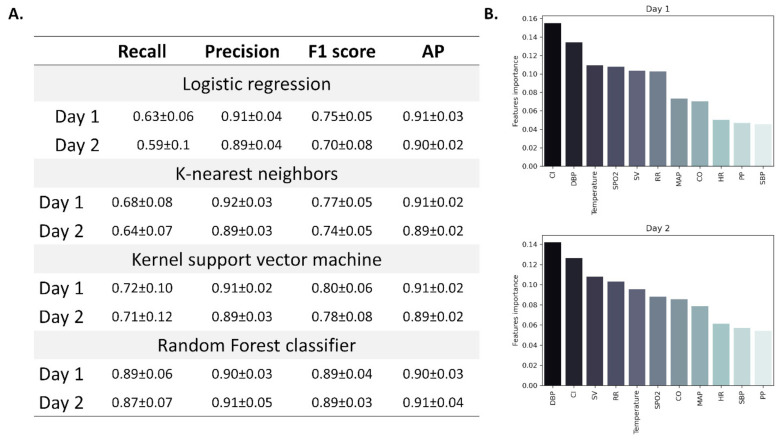
Classification models used for the prediction of viral infection based on changes in physiological parameters. Four models were tested for viral infection classification: logistic regression, k-nearest neighbors, kernel support vector machine, and random forest. Models’ performance was assessed using several metrices (**A**). Panel (**B**) features importance as calculated for the random forest model in days one and two before infection. Data are presented as mean ± standard deviation. DBP: diastolic blood pressure; CI: cardiac index; SV: stroke volume; RR: respiratory rate; SPO_2_: blood oxygen saturation; CO: cardiac output; MAP: mean arterial pressure; HR: heart rate; SBP: systolic blood pressure; PP: pulse pressure.

According to the lab tests and survey results, we defined the third day as the day in which the disease was most definite in terms of severity (the combination of higher viral load together with complaints). Therefore, we tested whether we can use the measured physiological parameters in the first and second days after exposure to classify the participants as flu-positive or negative before any major symptoms appear (trying to define changes during the pre-symptomatic phase). Accordingly, mean changes from baseline measurements were calculated for each participant, for both days and used as features in the model. Since the data is imbalanced (*n* = 11 and 105 for asymptomatic and symptomatic participants, respectively), we used a common method (SMOTE, see methods) to randomly scale up the asymptomatic cases. Among the four models that were tested (logistic regression, k-nearest neighbors, kernel support vector machine, and random forest), random forest achieved the highest overall score (Figure 6A). Using random forest classification, we were able to classify participants as flu-positive or negative with an F1 score (the harmonic mean of precision and recall) of 0.89 ± 0.04 and 0.89 ± 0.03 by using data from the first and second days, respectively. Avarage precision (AP, weighted mean of precision-recall curve) was slightly greater when using data from the second day (0.91 ± 0.04) (Figure 6A). Cardiac index and diastolic blood pressure had the greatest importance when using data from the first (0.155 and 0.134, respectively) and second day (0.126 and 0.142, respectively) (Figure 6B), with both found as the best predictors of disease onset.

## 4. Discussion

In this study, we show that frequent monitoring using the wearable remote monitoring platform may be used in clinical studies by providing advanced clinical insights previously not available. Moreover, collection of multiple physiological parameters is of clinical importance, not only within the scope of clinical studies but also in the daily lives of individuals. As early detection of a biological outbreak is a major mission of national health systems worldwide, as recently highlighted in the COVID-19 pandemic, this tool could allow for improved response due to its automatic, continuous, and real-time collection and transmission of objective physiological data. Currently-used bio-surveillance systems provide important epidemiological data. However, it is still difficult to rely on this data for early situational awareness as this data is acquired in hindsight and usually, after an outbreak of a disease is in advanced stages. In many cases, it includes mainly subjective information based on questionnaires completed by participants.

We assumed that following treatment with the vaccine or the placebo, we will have two distinct groups of participants. Since the novel H3N2 vaccine did not confer protection against the virus, the results did not show such a distinct difference between the two groups.

When comparing the MVA-treated to the placebo-treated individuals, we see that with most physiological parameters, there were no differences between the groups. Differences were seen in diastolic blood pressure and in mean arterial pressure, with the placebo-treated group showing an increase in these vitals when compared to the baseline levels. Since the vaccine was not effective, we cannot tell whether this observation is the result of the effect of the vaccine.

Importantly, though no time-symptom interactions were found, and similar trends were observed in both groups, the majority of changes reached significance only among participants in the symptomatic group.

SpO_2_, RR, HR, and temperature are regarded as relevant vital signs in the case of influenza. However, it is well accepted that they are not sensitive enough to allow detection, especially during the early phase of the infection [13]. We show here that a set of advanced physiological parameters, some of which were not monitored in previous studies due to technological limitations, could provide an early warning of infection. The elevation in SV, CO, and CI along with the reduction in SVR following the inoculation might provide additional insights in our understanding of the natural history of flu.

As mentioned, most of the physiological changes appeared in the cardiovascular system, and did not correlate with the severity of the upper respiratory tract complaints mentioned by the participants. In addition, while no changes or minor changes (up to 0.3%) were observed in SpO_2_ and RR, changes in advanced cardiovascular parameters (HR, 5 9%; SBP, 1.5%; DBP, 2%; MAP, 2%; SV, 4%; CO, 10%; CI, 12%; SVR, 10%) were apparent before any clinical reports.

By using the Random Forest classifier, we demonstrated how hemodynamic parameters, including DBP, CI, and SV can help distinguish between individuals that will develop full-blown clinical influenza and those that will not show clinical signs and symptoms.

When analyzing the data and comparing between individuals in which the virus was detected and individuals in which the virus was not detected, we see distinct differences, strengthening the notion that changes are more related to the viral load than to the treatment provided.

Physiological changes started to appear before the individuals had complaints and symptoms, and reached their peak on the third day after exposure. The peak of physiological changes appeared when the subjects only started to complain and show symptoms, and with a concurrent increase in viral load, emphasizing that these processes appear at the same time and show high correlation. This is a strong indication that early-stage monitoring may potentially serve as an early indicator of infection, improve situational awareness, and allow for early use of interventional measures such as isolation and medical treatment.

We found differences in the extent of physiological changes among the groups, with the middle age group showing increased changes when compared to the young, and males showing increased changes when compared to women. This might have significance when trying to better define the natural history of influenza infection in humans, especially concerning early detection and treatment adjustments.

Defining a pattern of change among these physiological parameters might enable us to predict and detect early influenza infections in the future, thus helping health care systems to battle a potential pandemic.

### Limitations

Since the vaccine was found to be ineffective, the majority of participants were flu-positive, which affected the ability of classification models to identify flu-negative participants.

These results from high numbers of flu-positive participants vs. flu-negative limits direct adoption to real-world scenarios. Further balanced studies could allow better insights into the sensitivity and specificity of this tool. Though even a high number of false positives could still be clinically relevant in the case of an outbreak, we should be cautious in this specific study, since we had a small number of participants, especially flu-negative.

The cohort included in this study was limited in size and in individuals’ characterizations. A larger cohort with a wider range of age and BMI is needed to substantiate the results, improve our understanding of the physiological changes seen before and during influenza, and better represent the US population. Moreover, this was not a field study, rather a well-controlled trial within a confined facility to assure flu would not spread, making it difficult to simulate a real-world scenario. In contrast, it was easier to perform the exposure procedure and collect the data, showing that prolonged monitoring of subjects using a remote patient monitoring platform is feasible. Therefore, the results could serve as a starting point for future studies of this topic, with additional research in more balanced classes in all mentioned aspects. This could ultimately enable a better performance of the model and provide better tools for early pre-symptomatic detection of flu.

As more and more individuals are using wearable devices in the everyday life, and the quality of these devices, as well as the number of variables measured, keeps increasing, it could be estimated that in the future, more people will be using such devices that could help with early detection of outbreaks among the general public [32].

Similar studies would allow us to better differentiate the patterns we found with flu, from other causes of disease, improving its applicability in real-world settings.

## 5. Conclusions

Continuously monitoring advanced hemodynamic parameters could help in defining early pre-symptomatic changes during the early stages of influenza. This has the potential to improve future bio-surveillance efforts by providing early detection of outbreaks, improving situational awareness, and enabling better use of isolation measures and medical treatments.

## Figures and Tables

**Table 1:** General characteristics of the participants, and average baseline values of the physiological parameters collected during the study. NS, not significant.

Characteristic	Flu-Positive (*n* = 105)	Flu-Negative (*n* = 11)	*p*-Value	All (*n* = 116)
Age (Years)	39.3 ± 12.0	40.1 ± 10.6	NS	40.2 ± 10.5
Sex (M/F)	46/59	3/8	NS	49/67
Respiratory rate (breath/min)	13.3 ± 0.7	13.2 ± 1.2	NS	13.2 ± 2.3
SpO_2_ (%)	96.7 ± 1.2	96.5 ± 1.5	NS	96.5 ± 2.9
Heart rate (beats/min)	66.2 ± 8.4	66.6 ± 7.0	NS	66.6 ± 12.7
Systolic blood pressure (mmHg)	126.0 ± 10.7	124.4 ± 9.5	NS	124.7 ± 11.4
Diastolic blood pressure (mmHg)	77.2 ± 10.6	75.2 ± 8.4	NS	75.4 ± 10.1
Mean arterial pressure (mmHg)	93.5 ± 10.1	91.6 ± 8.2	NS	91.8 ± 10.0
Pulse pressure (mmHg)	48.8 ± 7.0	49.2 ± 6.2	NS	49.3 ± 7.2
Stroke volume (mL)	84.3 ± 10.6	83.3 ± 11.5	NS	83.1 ± 13.3
Cardiac output (L/min)	5.6 ± 1.2	5.6 ± 0.9	NS	5.6 ± 1.6
Cardiac index (L/min/m^2^)	3.3 ± 0.7	3.3 ± 0.6	NS	3.3 ± 1.0
Systemic vascular resistance (dynes·s·cm^−5^)	1400.8 ± 214.0	1411.7 ± 316.1	NS	1415.2 ± 388.5
Temperature (°C)	36.3 ± 0.2	36.0 ± 1.8	NS	36.0 ± 2.3

## Data Availability

The data that support the findings of this study are available on request from the corresponding author (A.E.). The data are not publicly available due to definitions of the funding authority.
